# Stereotactic body radiotherapy with a focal boost to the MRI-visible tumor as monotherapy for low- and intermediate-risk prostate cancer: early results

**DOI:** 10.1186/1748-717X-8-84

**Published:** 2013-04-09

**Authors:** Shafak Aluwini, Peter van Rooij, Mischa Hoogeman, Wim Kirkels, Inger-Karine Kolkman-Deurloo, Chris Bangma

**Affiliations:** 1 Department of Radiation Oncology, Erasmus MC-Daniel den Hoed Cancer Center, Groene Hilledijk, Rotterdam, The Netherlands; 2 Departmentv of Urology, Erasmus MC-Daniel den Hoed Cancer Center, Groene Hilledijk, Rotterdam, The Netherlands

**Keywords:** Clinical outcome, Low- and intermediate-risk, Radiotherapy, Prostate cancer, Stereotactic body radiation

## Abstract

**Background:**

There is growing evidence that prostate cancer (PC) cells are more sensitive to high fraction dose in hypofractionation schemes. High-dose-rate (HDR) brachytherapy as monotherapy is established to be a good treatment option for PC using extremely hypofractionated schemes. This hypofractionation can also be achieved with stereotactic body radiotherapy (SBRT). We report results on toxicity, PSA response, and quality of life (QOL) in patients treated with SBRT for favorable-risk PC.

**Methods:**

Over the last 4 years, 50 hormone-naïve patients with low- and intermediate-risk PC were treated with SBRT to a total dose of 38 Gy delivered in four daily fractions of 9.5 Gy. An integrated boost to 11 Gy per fraction was applied to the dominant lesion if visible on MRI. Toxicity and QoL was assessed prospectively using validated questionnaires.

**Results:**

Median follow-up was 23 months. The 2-year actuarial biochemical control rate was 100%. Median PSA nadir was 0.6 ng/ml. Median International Prostate Symptoms Score (IPSS) was 9/35 before treatment, with a median increase of 4 at 3 months and remaining stable at 13/35 thereafter. The EORTC/RTOG toxicity scales showed grade 2 and 3 gastrointestinal (GI) acute toxicity in 12% and 2%, respectively. The late grade 2 GI toxicity was 3% during 24 months FU. Genitourinary (GU) grade 2, 3 toxicity was seen in 15%, 8%, in the acute phase and 10%, 6% at 24 months, respectively. The urinary, bowel and sexual domains of the EORTC-PR25 scales recovered over time, showing no significant changes at 24 months post-treatment.

**Conclusions:**

SBRT to 38 Gy in 4 daily fractions for low- and intermediate-risk PC patients is feasible with low acute and late genitourinary and gastrointestinal toxicity. Longer follow-up preferably within randomized studies, is required to compare these results with standard fractionation schemes.

## Background

Although external beam radiotherapy (EBRT) is a highly effective treatment for prostate cancer (PC), the long course of 7–9 weeks can have a negative impact on the patients’ quality of life (QoL) and hospital resources. Hypofractionated radiotherapy is used increasingly because of its radiobiological benefits, acceptable toxicity, economic and social advantages.

Several publications suggest a radiobiological rationale for hypofractionated radiotherapy in PC [1,2]. This indicates a high sensitivity of PC to fraction dose but not to the total dose, suggesting the possibility of significant therapeutic benefit from hypofractionation in terms of local control and reduction of normal tissue complication probability for bladder and rectum [1-3].

Brachytherapy is commonly used as treatment for PC because of the possibility to deliver a high dose to the prostate while sparing the surrounding organs at risk (OARs). The use of high-dose-rate brachytherapy (HDR-BT) is proven to be safe and effective and this technique is increasingly used either as a boost after EBRT or as monotherapy [4,5]. Fuller et al. [6] demonstrated that it is possible to achieve the same dose distributions with SBRT as with HDR-BT. Based on these findings and our HDR-BT experience, we initiated an SBRT protocol to treat low- and intermediate-risk PC patients. This protocol was used for patients who were not eligible for HDR brachytherapy.

## Methods

### Patients and planning

Between June 2008 and November 2011, 50 hormone-naïve patients with biopsy-proven low- to intermediate- risk PC underwent SBRT treatment of PC, using the Cyberknife®, in four daily fractions of 9.5 Gy to a total dose of 38 Gy.

The first 10 patients were treated in a pilot study with the results reported in 2010 [7]. The inclusion criteria can be found in this report as well. These patients were not eligible for HDR brachytherapy because of a large volume of the prostate (> 50 cc), or a combination of limited urine flow/second (Q-max < 10 ml/sec.) and a significant residual volume in the bladder (>100 cc) (37/50, 74%). Other reasons were: transurethral resection of the prostate in the medical history in six patients (12%), pelvic surgery in two (4%) and hip joint prostheses in five (10%). Patients with clinical stages T1c-T2a, Gleason-score 6 and PSA ≤10 ng/ml were defined as low-risk PC. Patients with PSA 10–20 ng/ml, and/or T2b-T3a and/or Gleason-score 7, were defined as intermediate-risk PC [8].

In all patients, four gold fiducial seeds were implanted in the prostate through ultrasound-guided trans-perineal pre-loaded needles. One week after fiducial implantation, computed tomography (CT) and magnetic resonance images (MRI) were acquired. T1- and T2-weighted sequences were performed (1.5 Tessla without endorectal coil) to elaborate the treatment plan after placement of a Foley catheter. The Foley catheter was delineated as the urethra. The CT and MRI images were matched on the markers and the Foley catheter.

All patients followed a low fiber dietary protocol to minimize intestinal activity.

The MultiPlan (version 2.1.5, Accuray) treatment planning system was employed. If the dominant tumor was visible on the MRI, an integrated boost to the visible tumor was planned up to 11 Gy/fraction which is 120% of the prescribed dose (PD). The planning target volume (PTV) included the prostate expanded by 3 mm in all directions and had to receive ≥ 95% of the PD. Minor violation of the constraints up to 110% of the constraint dose were accepted. Details on treatment planning and applied constraints can be found in an earlier report [7].

### PSA measurement, toxicity, and QoL

All patients were followed prospectively. Biochemical failure (BF) was determined according to the Phoenix definition (nadir PSA + 2 ng/ml) [9]. A PSA bounce is defined as a transient rise in the PSA level with a subsequent normalization of the PSA values [10]. GI and GU toxicity was defined and reported using the Radiation Therapy Oncology Group and European Organization for Research and Treatment of Cancer (RTOG-EORTC) scoring criteria [11]. RTOG-EORTC toxicity and the IPSS questionnaires were sent to the patients at the following time points: baseline, at 1, 2, 3, 6, 12 months after treatment, and twice yearly thereafter. International Index of Erectile Function (IIEF) and the EORTC QLQ-PR25 questionnaires were sent at baseline, 6 and 12 months after treatment, and yearly afterward. All patients were seen every 3 months in the first year and subsequently twice yearly.

### Statistical analysis

The GI and GU toxicity were evaluated according to the EORTC-RTOG toxicity scores, using a combination of the patients’ questionnaires and physicians’ charts. The highest-score of toxicity was recorded. Toxicity within 90 days after radiotherapy was considered acute toxicity, and toxicity after 90 days was considered late toxicity. The IPSS, the PR-25 and the IIEF questionnaires were used to asses the GU, GI functional QoL, and erectile function. These questionnaires were analyzed to obtain the net effect on function compared to baseline.

## Results

Table 1 shows patient characteristics. The MRI staging was: T1c (9, 18%), T2a (22, 44%), T2b (4, 8%), T2c (2, 4%), T3a (13, 26%). The mean dosimetric constraints were mostly met but in 30% a minor violation was accepted.

**Table 1:** Patient, tumor and treatment characteristics

		**n**	**n%**	**Mean (min.-max.)**
Age				68 (48–80)
Fup (months)				23 (9–47)
TNM	T1cN0	31	62%	
	T2aN0	17	34%	
	T2bN0	1	4%	
	T2cN0	1	2%	
Gleason	3+3	41	82%	
	3+4	9	18%	
IPSA				8.2 (1.3–16)
Prostate volume				48 (22–110)
Q-max				13 (4–33)
Residual				87 (0–300)
Risk Group	Low risk	30	60%	
	Intermediate risk	20	40%	
Position positive biopsy	Single sided	31	62%	
	Double sided	19	38%	
Count positive biopsy	1	12	24%	
	2	14	28%	
	3	9	18%	
	4	8	16%	
	5	5	10%	
	7	2	4%	

In 14 patients (28%), a visible dominant tumor was detected on the contrast-enhanced MRI with a mean tumor volume of 1.2 cc (range, 0.46–4.1 cc). In three patients more than one lesion was detected. The mean dose to this visible dominant tumor area defined as gross target volume (GTV) was 47.8 Gy (40.3–53.8 Gy) which is 120–150% higher than the PD. Capsule invasion on T2-weighted MRI was registered in 13 (26%) patients. The area of invasion was included in the high dose area (> 100 PD) without changing the margin used.

The treatment time was between 55–130 minutes, there was no difference between patients with or without boost with a mean of 64 minutes versus 59 minutes, respectively.

All patients were alive without biochemical failure at the end of follow-up.

### Acute toxicity

The mean IPSS before treatment was 9/35, did not increase in the acute phase. The percentages of grade 2 and 3 acute GI toxicity were 12% and 2%, respectively. The incidence of grade 2 and 3 acute GU toxicity was 15% and 8%, respectively.

The most common GU complaints during this phase were urinary urge and increased night voiding frequency. Increased stool frequency was the main GI complaint. One out of the first 10 patients needed an indwelling bladder catheter because of urinary retention 1 week after completion of the radiation course. This patient had a baseline prostate volume of 110 cc; the maximum prostate volume allowed was lowered to 90 cc in our protocol following this incident.

### Late toxicity

**Figure 1:**
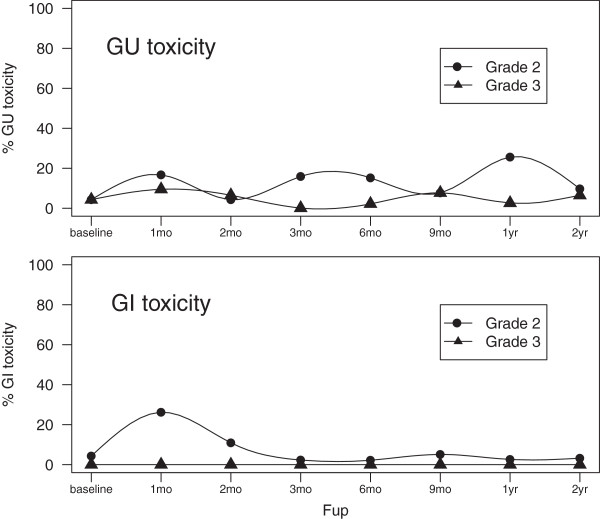
**GI and GU.** Gastrointestinal and Genitourinary toxicity (%).

**Figure 2:**
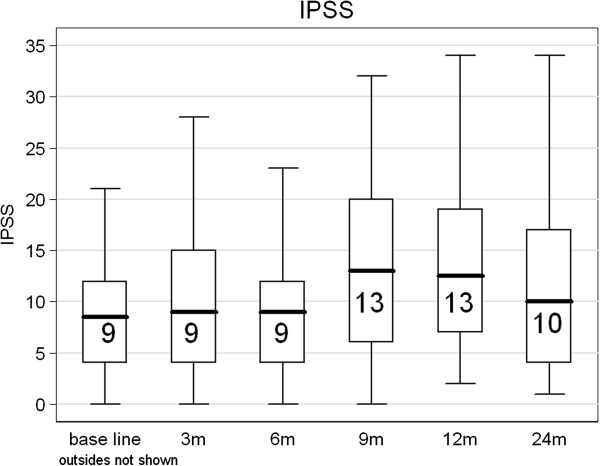
**IPSS.** International Prostate Symptoms Score (IPSS) changes (mean).

The chronologic incidence of grade ≥ 2 GI and GU toxicity is shown in Figure 1. The mean IPSS increased to 13/35 at 12 months after treatment, resolved to 10/35 at 24 months (Figure 2). The main cause of grade 2 GU toxicity was increased night voiding frequency (> 4×/night). This reached a peak at 12 months in 20% of patients resolving to 10% at 24 months. Other complaints were urge and radiation prostatitis in two patients which was treated by NSAID. The GI toxicity was limited to increased stool frequency and necessity of using adult diapers which resolved by all (2) patients within 6 months.

There were no differences in toxicity between the group patients with MRI-visible tumor receiving a boost in comparison to the others without MRI-visible tumor.

### PSA nadir and bounce

**Figure 3:**
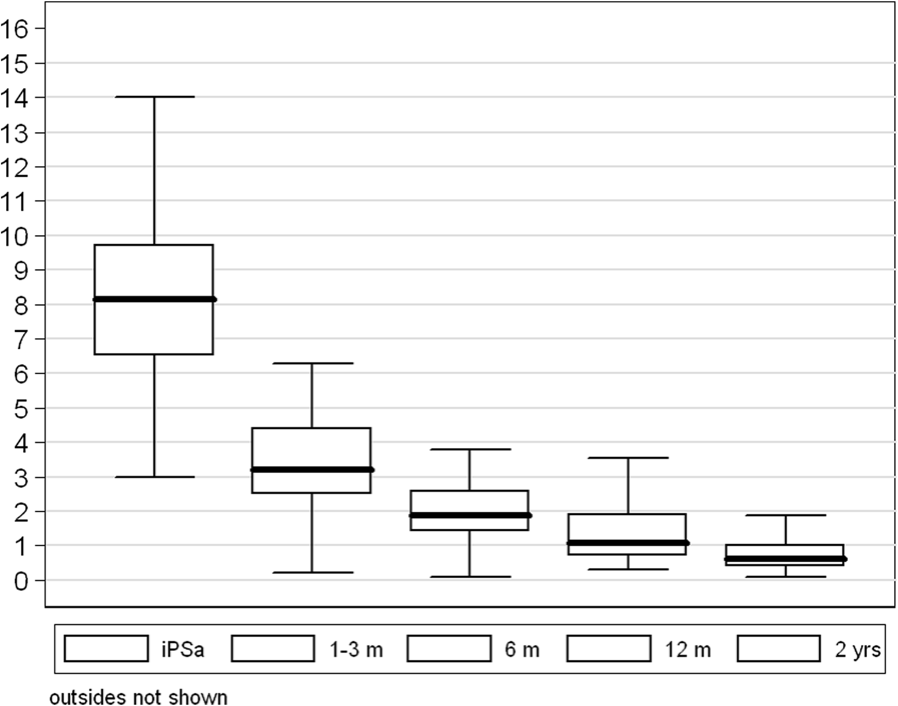
**PSA.** PSA response (outsides not shown).

The median PSA nadir for patients with a follow-up ≥ 24 months was 0.6 ng/ml (range, 0.1–2 ng/ml) and 1.1 ng/ml for patients with a FU ≥ 12 months (Figure 3). Nadir PSA < 1 was reported in 27 patients (59%). PSA bounce was recorded in seven patients (14%), and the mean interval to the bounce episode was 12 months (range, 4.0–22 months).

### QoL

**Figure 4:**
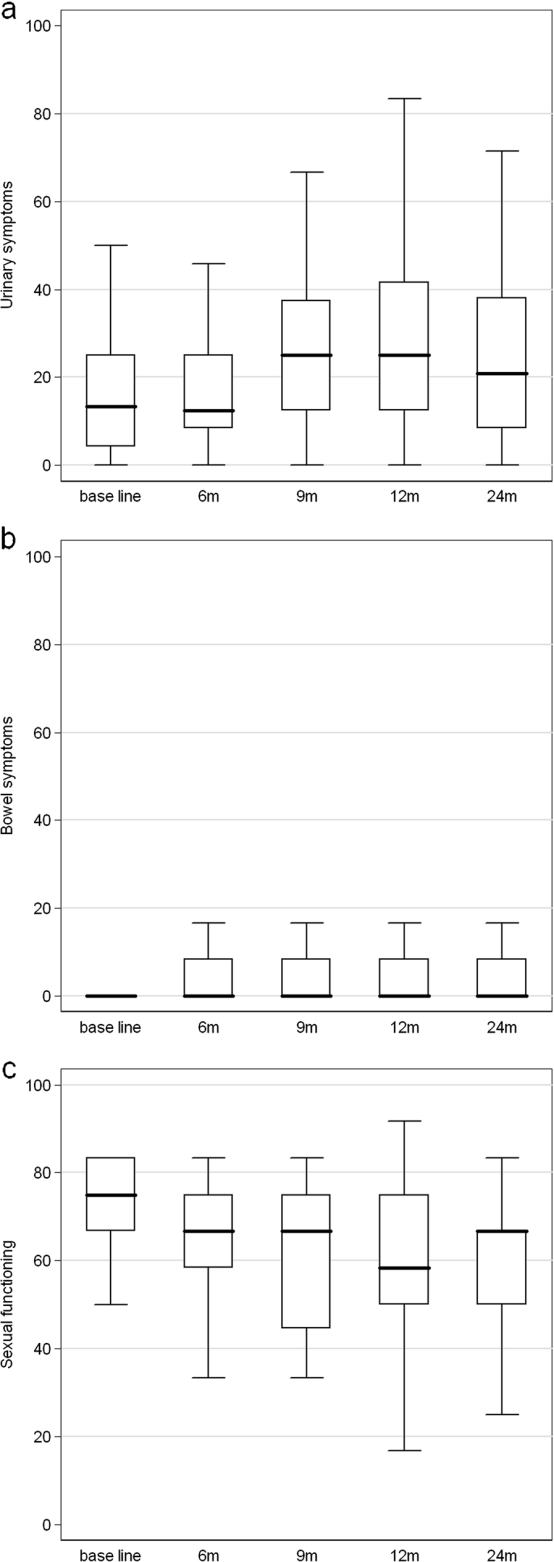
**PR-25.** EORTC QLQ-PR-25 changes (mean). **a**: urinary symptoms, **b**: bowel symptoms, **c**: sexual function.

The mean changes in the EORTC-QLQ PR25 score for each domain are shown in Figure 4. The median PR-25 GU score was increased from 13 before the treatment to 25 at 12 months returning to 21 after 2 years (*p*=0.264). The median bowel symptoms did not change after the treatment. The sexual function was decreased from 75 at the baseline to 66.76 after 2 years (*p=0.145*). The incontinence and bother score was slightly and insignificantly increased in the first 12 months post-treatment, returning to normal afterward. The IIEF results with only 24 months FU are not yet mature for publication [12].

## Discussion

SBRT is increasingly used because of the possibility of this image-guided technique to minimize the margins needed for treatment, reducing normal tissue dose and resulting in a lower percentage of toxicity. This, in combination with the possibility of SBRT to deliver a high radiobiological dose in few fractions makes this technique ideal for the treatment of PC. Our fractionation scheme was used in HDR-brachytherapy series with excellent 10-year results [5]. Despite such good results, the invasive character and the need of hospitalization and anesthesia makes brachytherapy less convenient and a labor intensive method.

### PSA response

In our patients, an excellent early PSA nadir was achieved. This is comparable to HDR and EBRT series with longer FU [5,13]. This is important because the PSA nadir could predict long-term BF and distant metastases-free survival [5,13,14]. Because of our short FU, the final nadir may not yet been reached. Other SBRT series have reported lower PSA nadirs after longer follow-up [15,16]. The percentage of patients with bounce phenomena was lower than the percentage in brachytherapy and EBRT series [4,10]; the short FU may explain this.

### Toxicity

The toxicity percentage was conform literature. Although this group had more GU complaints because of their predisposing factors (large volume, bad flow, high IPSS, TURP or abdominal surgery) before the treatment, they did not show more toxicity in the acute neither in the late phase. This suggests that our treatment could reach lower toxicity percentages in patients without these predisposing factors as confirmed in other SBRT series; e.g. Freeman et al. [15] reported 7% and 2.5% grade 2 and 3 GU toxicity, respectively with 2.5%, 0% grade 2, 3 GI toxicity, respectively. King et al. used the same fractionation scheme (5 fractions) as Freeman [16] and reported 5% and 3.5% for grade 2 and 3 GU late toxicity, respectively. The grade 2 GI toxicity was reported in only 2% of the patients. Several series reported toxicities between 3% and 20% [17-19].

The treatment time was relatively high comparing with conventional series ( > 55 minutes). Fowler. et al. [20] mentioned the influence of treatment time for high fraction dose on the log cell kill (BED) suggested a decrease in the BED for fraction duration of more than 30 minutes. Although this subject requires more discussion it may play a role in decreasing late toxicity for this regimen.

Using our fractionation scheme Jabbari et al. [21] reported a higher grade 2 late toxicity, but a lower grade 3 late toxicity. The number of patients treated with monotherapy was only 20 patients with a shorter follow-up. To date Jabbari published the only series using our HDR like 4×9.5 Gy scheme.

The relative higher grade 3 GU toxicity in our series compared to other SBRT series could be explained by patient selection; we treated patients with more complaints and predisposing factors. We also used the combination of questionnaires and physician’s charts reporting the highest score from both, which could result in higher scores. The difference in measurement instrument using the EORTC/RTOG criteria [7,15-17] in our group where some other series used the Common terminology Criteria for adverse events (CTCAE) [18,19,21,22], makes a comparison between series difficult.

The different dose levels and fractionation schemes between series could also be a reason for differences in toxicity records Table 2 shows toxicity of published SBRT series.

**Table 2:** Toxicity SBRT published series

	**N patients**		**FU**	**Acute GU**		**Late GU**		**Acute GI**		**Late GI**	
				**Grade 2**	**Grade 3**	**Grade 2**	**Grade 3**	**Grade 2**	**Grade 3**	**Grade 2**	**Grade 3**
			**months**	**%**	**%**	**%**	**%**	**%**	**%**	**%**	**%**
Townsend 2011 [19]	48	5×7–7,25		10	8			13	0		
King 2012 [16]	67	5×7.25	31			5	3			2	0
Freeman 2011 [15]	41	5×7–7,25	55			7	2.5			0	0
Katz 2010 [17]	304	5×7–7,25	30	4.7	0	2.9	0	3.6	0	2.9	0
Madsen 2010 [22].	40	5×6.7	60	20.5	2.5	12.5	2.5	13	0	7.5	0
Jabbari 2012 [20]	38	4×9.5	18	45	0	8	5	5	0	3	0
This report	50	4×9.5	24	15	8	10	6	12	2	3	0

### QoL

The EORTC-QLQ PR25 questionnaire is a validated 25-item instrument with four domains (urinary, bowel, sexual, and hormonal), as well as two urinary subscales of incontinence and irritative/bother [23]. Responses are transformed to a scale of 0–100. For functional scales, higher scores represent better QoL. For symptom scales, higher scores indicate more symptoms or more problems. In our cohort, there was a significant increase in the urinary symptoms in the first year which was reversed at 24 months. The bowel symptoms did not increase and remained stable during the 24 months after treatment, which indicates limited bowel toxicity. The sexual function changes were more obvious at 12 months but not significant after 24 months. This has to be confirmed with the results of the IIEF-questionnaires. There is very limited data published regarding sexual function after SBRT for PC. The group of King et al. [12] reported the sexual function during 3 years FU of 32 PC patients having undergone SBRT with 5 fractions of 7.25 Gy. They used the Expanded Prostate Cancer Index Composite (EPIC) [12], unfortunately with the same limitations as the PR-25 questionnaires to give a detailed analysis about the effect of treatment on the sexual function.

### SBRT

Published results of SBRT as monotherapy reported a short FU and many variations in fraction size, total dose, and technique used. Also, the toxicity was measured with different tools making comparison difficult. The group of Freeman and King used five fractions of 7–7.25 Gy first in daily fractions but later in every-other-day fractions [15,16]. They planned a more homogeneous dose distribution and used less strict constraints. This is in contrast to our technique, which was also used by Jabbari [21] administering four daily fractions of 9.5 Gy, where the dose distribution inside the prostate is heterogeneous up to 40% above the PD. This heterogeneity contributes to a higher dose in the entire prostate which, in the light of the low alpha/beta of the prostate, may contribute to the excellent HDR brachytherapy results. Furthermore, this gives us the opportunity to shape the dose distribution in the critical area of the prostate to giver higher dose in the entire peripheral zone of the prostate where almost 65% of prostate tumors were found in prostatectomy specimens [24]. Our constraints for bladder and rectum were the same as that of our HDR brachytherapy, restricting the volumes receiving 80% of the PD to <1.5 cc. Minor violations for the rectum and bladder constraints were accepted (80% PD to 1.5–2 cc) in 30% of the patients, according to the position of the tumor and the patient’s anatomical variation, as more than 50% of this group had a prostate volume > 50 cc with a prominent transient zone.

King et al. reported more rectal toxicity in the daily treated group versus the every-other-day treated group [16]. In our current cohort, 10 patients were treated with a weekend rest of 2 days between the four fractions due to logistic reasons. These 10 patients did not show a lower rectal or bladder toxicity. We are aware that the limited number of patients and the relatively short FU make it hard to reach a conclusion about this point.

The number of patients with a visible tumor on the MRI was low (28%), this could be explain because of the inclusion of more low-risk patient with Gleason-score of 6 which is not always visible on the MRI.

SBRT is an emerging treatment approach for PC and so far has been safe and effective as monotherapy. However trials are warranted addressing many of the raising questions about the optimal fraction dose, total dose, safety constraints and the optimal technique to be used. Recently, the results of a phase 1 study concerning dose escalation and toxicity has been published [25]. Next year we will start a phase III trial to compare this SBRT schemes with the standard EBRT of 39×2 Gy to address outcomes, toxicity and QoL.

## Conclusions

In this cohort where many patients were not suitable for HDR brachytherapy, an SBRT regimen of four daily fractions of 9.5 Gy shows low toxicity in line with the published literature. The PSA response to date is good without any BF. More patients and longer FU is needed to confirm this conclusion.

## Competing interests

The authors declare that they have no competing interests.

## Authors’ contributions

SA, PvR, MH, IK, CB have made substantial contributions to conception and design; SA, PvR, WK made substantial contributions to acquisition of data; PvR to the analysis of data; SA, PvR were involved in drafting the document; IK, WK, CB, MH Revised the document critically. All authors approved this version to be published. All authors read and approved the final manuscript.
